# Lipid nanostructures for antioxidant delivery: a comparative preformulation study

**DOI:** 10.3762/bjnano.10.174

**Published:** 2019-08-29

**Authors:** Elisabetta Esposito, Maddalena Sguizzato, Markus Drechsler, Paolo Mariani, Federica Carducci, Claudio Nastruzzi, Giuseppe Valacchi, Rita Cortesi

**Affiliations:** 1 Department of Chemistry and Pharmaceutical Sciences, University of Ferrara, I-44121 Ferrara, Italy; 2 Bavarian Polymerinstitute "Electron and Optical Microscopy" University of Bayreuth, Germany; 3 Dipartmento di Scienze della Vita e dell'Ambiente, Università Politecnica delle Marche, I-60131 Ancona, Italy; 4 NC State University, Plants for Human Health Institute, Animal Science Dept. NC Research Campus, Kannapolis, NC 28081, USA; 5 Department of Biomedical and Specialist Surgical Sciences, University of Ferrara, I-44121 Ferrara, Italy; 6 Department of Food and Nutrition, Kyung Hee University, Seoul, Korea

**Keywords:** α-tocopherol, cryogenic transmission electron microscopy (cryo-TEM), dermocosmetics, HO-1, nanostructured lipid carriers (NLCs), retinoic acid, skin pollution, solid lipid nanoparticles (SLNs)

## Abstract

This investigation is a study of new lipid nanoparticles for cutaneous antioxidant delivery. Several molecules, such as α-tocopherol and retinoic acid, have been shown to improve skin condition and even counteract the effects of exogenous stress factors such as smoking on skin aging. This work describes the design and development of lipid nanoparticles containing antioxidant agents (α-tocopherol or retinoic acid) to protect human skin against pollutants. Namely, solid lipid nanoparticles and nanostructured lipid carriers were prepared using different lipids (tristearin, compritol, precirol or suppocire) in the presence or absence of caprylic/capric triglycerides. The formulations were characterized by particle size analysis, cryogenic transmission electron microscopy, small-angle X-ray diffraction, encapsulation efficiency, preliminary stability, in vitro cytotoxicity and protection against cigarette smoke. Nanostructured lipid carriers were found to reduce agglomerate formation and provided better dimensional stability, as compared to solid lipid nanoparticles, suggesting their suitability for antioxidant loading. Based on the preformulation study, tristearin-based nanostructured lipid carriers loaded with α-tocopherol were selected for ex vivo studies since they displayed superior physico-chemical properties as compared to the other nanostructured lipid carriers compositions. Human skin explants were treated with α-tocopherol-loaded nanostructured lipid carriers and then exposed to cigarette smoke, and the protein levels of the stress-induced enzyme heme oxygenase were analyzed in skin homogenates. Interestingly, it was found that pretreatment with the nanoformulation resulted in significantly reduced heme oxygenase upregulation as compared to control samples, suggesting a protective effect provided by the nanoparticles.

## Introduction

Air pollution increasingly affects industrialized urban areas in a negative manner with dramatic consequences for the environment and human health. This problem also affects rural areas, worsening the air quality all over the world. Besides being the primary cause of many respiratory diseases (e.g., chronic obstructive pulmonary disease, asthma and lung cancer), pollution is also responsible for cutaneous pathologies, spanning from skin aging, inflammation and allergy to skin cancer [1].

Cigarette smoke (CS) is one of the major toxic pollutants, exerting an important role in the onset of many serious and fatal diseases. Indeed, it is well known that CS can provoke various pathologies especially related to the lungs (e.g., cancer, emphysema, bronchitis) as well as the cardiovascular apparatus [2–3]. In the last two decades, the noxious effect of CS on skin has been well demonstrated [4–6]. For instance, the chronic exposure of skin to CS induces premature skin ageing, delayed wound healing, psoriasis and inflammatory skin diseases [3]. CS increases the risk of squamous cell carcinoma, with respect to non-smokers, as well as oral leukoplakia and oral cancers, such as lip cancer*.* Indeed, tobacco smoke is constituted of thousands of toxic compounds, including benzene, formaldehyde, hydrogen cyanide, carbon monoxide, arsenic and radioactive components, producing free radicals that cause oxidative stress [7]*.* The release of reactive oxygen species from tobacco smoke provokes a series of systemic immunomodulatory effects that leads to a compromised inflammatory response. These destructive mechanisms also affect collagen synthesis and the skin cellular reparative effects [8–9]. It has been found that antioxidants play a key role in the regulation of the deleterious activity exerted by CS in humans, nevertheless CS alters the requirements of antioxidants, such as vitamins E and A [9–14]. In this respect, quitting smoking does not always resolve the issue, since even more toxic effects have been shown from exposure to second-hand smoke.

Recently many cosmetic producers have focused their efforts towards antipollution dermocosmetics that are able to defend the skin against prolonged and repetitive daily exposure to pollutants; for instance, film-formers or skin rejuvenating excipients have been developed. Nevertheless, these strategies offer merely a short-term improvement of skin barrier function. Thus, in this respect, there is an unmet need for an effective product that endows skin protection from pollutants from long-term exposure, as well as for antipollution test methods suitable for assessing product efficacy and safety [15].

Vitamin E is a potent antioxidant, able to counteract the reactive oxygen species production during fat oxidation and free radical propagation – indeed it can protect the cell membranes from free radical attack, acting against lipid peroxidation. Vitamin E exists in 8 different forms, 4 tocopherols and 4 tocotrienols [16]. Among them α-tocopherol (TOC) can be mostly adsorbed and accumulated, thus it is largely employed as an antioxidant for edible oils and in anti-aging products. Notably, TOC has been proposed for the treatment of cancer and skin barrier improvement [17–19].

Vitamin A is defined as a group of lipophilic retinoids, including retinoic acid (RA), derived from food and stored in the liver. Due to its antioxidant action, RA plays a role in cancer chemoprevention and differentiation [20]. Particularly, RA has been proposed in the treatment of breast, lung and liver cancers [21–22]. Notably, it has been demonstrated that CS induces RA deficiency [23].

Despite the enormous potential of TOC and RA, some drawbacks are associated to their topical use, such as photodegradation, poor water solubility and irritative skin effects when employed in high dosage [24–25]. Thus, TOC and RA need to be loaded in specialized formulations suitable for skin application and able to adequately protect them from degradation. In this respect, recently different lipid nanoparticles have been proposed, including solid lipid nanoparticles (SLNs) and nanostructured lipid carriers (NLCs) [26–28]. SLNs possess several advantages over conventional lipid formulations being able to carry drugs in a biocompatible solid nanometric matrix, thus achieving

1. improvement of solubility,
2. stability of the loaded active molecule and
3. suitability of administration through different routes [29–30].

NLCs represent a smart generation of lipid nanoparticles, being based on a blend of solid and liquid lipids that creates a disordered nano-matrix, able to load higher amounts of lipophilic molecules than SLNs and avoiding leakage during storage [31–34].

The choice of the type and concentration of the nanoparticle lipid matrix is crucial since it can affect the physico-chemical aspects of SLNs and NLCs, the encapsulation parameters, as well as the long-term stability of the formulation. Thus, in view of an industrial production, a preliminary formulation screening appears imperative [35–36].

The present investigation has been conducted to develop a nanoparticulate approach for counteracting skin pollution. In particular, a preformulation study was performed to select the type and composition of lipid nanoparticles suitable for encapsulation of TOC and RA. To assess the effect of antioxidant loaded in nanoparticles, a Western blot analysis has been performed to evaluate heme oxygenase expression on human skin explants treated with nanoparticles and exposed to CS.

## Experimental

### Reagents

The copolymer poly(ethylene oxide) (80)–poly(propylene oxide) (27) (poloxamer 188) was a gift from BASF ChemTrade GmbH (Burgbernheim, Germany). Miglyol 812 N, caprylic/capric triglycerides (miglyol) was a gift of Cremer Oleo Division (Witten, Germany). Glyceryl distearate (precirol ATO5, precirol), glyceryl dibehenate (compritol 888ATO, compritol) and mono-, di-, tri-glyceride esters of fatty acids (C_10_–C_18_) (suppocire AM, suppocire) were kind gifts of Gattefossè (Milan, Italy). Glyceryl tristearate (tristearin), α-tocopherol (TOC), retinoic acid (RA) and HPLC solvents were purchased from Sigma-Aldrich, Merck (Darmstadt, Germany).

### Preparation of lipid nanoparticles

Lipid nanoparticles were prepared by a hot homogenization technique based on ultrasound treatment. In both cases the dispersing phase was an aqueous solution of poloxamer 188 (2.5% w/w) [37]. In the case of SLN the disperse phase was constituted of one solid lipid (i.e., tristearin, precirol, compritol or suppocire), while in the case of NLC, a mixture between one solid lipid and the liquid lipid caprylic/capric triglycerides (miglyol) (1:1 w/w ratio) was employed. In both cases the lipid phase was 5 or 10% by weight, with respect to the whole weight of the dispersion. The nanoparticle dispersion acronyms and compositions are reported in Table 1 and Table 2.

**Table 1:** Composition of solid lipid nanoparticles (SLNs).

preparation	composition % (w/w)					
	lipid phase				water phase	
	tristearin	compritol	precirol	suppocire	poloxamer	water

SLN T5	5	–	–	–	2.37	92.63
SLN T10	10	–	–	–	2.25	87.75
SLN C5	–	5	–	–	2.37	92.63
SLN C10	–	10	–	–	2.25	87.75
SLN P5	–	–	5	–	2.37	92.63
SLN P10	–	–	10	–	2.25	87.75
SLN S5	–	–	–	5	2.37	92.63
SLN S10	–	–	–	10	2.25	87.75

**Table 2:** Composition of nanostructured lipid carriers (NLCs).

preparation	composition % (w/w)						
	lipid phase					water phase	
	tristearin	compritol	precirol	suppocire	miglyol	poloxamer	water

NLC T5	2.5	–	–	–	2.5	2.37	92.63
NLC T10	5.0	–	–	–	5.0	2.25	87.75
NLC C5	–	2.5	–	–	2.5	2.37	92.63
NLC C10	–	5.0	–	–	5.0	2.25	87.75
NLC P5	–	–	2.5	–	2.5	2.37	92.63
NLC P10	–	–	5.0	–	5.0	2.25	87.75
NLC S5	–	–	–	2.5	2.5	2.37	92.63
NLC S10	–	–	–	5.0	5.0	2.25	87.75

Firstly, an emulsion was obtained adding the poloxamer 188 aqueous phase (4.5/4.75 mL) heated at 80 °C to the molten lipid phase (250/500 mg), followed by mixing at 15000 rpm, at 80 °C for 1 min (IKA T25 digital ultraturrax). Secondly, the emulsion was subjected to ultrasound homogenization at 6.75 kHz for 15 min (Microson ultrasonic Cell Disruptor-XL Minisonix) and allowed to cool at 25 °C. Lipid nanoparticle dispersions were stored at room temperature. In the case of drug-loaded nanoparticles, TOC (0.4–0.8% w/w with respect to the whole dispersion; 8% w/w with respect to the lipid phase) or RA (0.02% w/w with respect to the whole dispersion; 0.4% w/w with respect to the lipid phase) were solubilized in caprylic/capric triglycerides (miglyol) and then added to the fused lipid phase before the emulsification step. The nanoparticle acronyms are reported in Table 3.

**Table 3:** Composition of antioxidant-containing NLCs.

preparation	composition % (w/w)								
	lipid phase							water phase	
	tristearin	compritol	precirol	suppocire	miglyol	TOC^a^	RA^b^	poloxamer	water

NLC T5-TOC	2.5	–	–	–	2.5	0.4	–	2.37	92.23
NLC T10-TOC	5.0	–	–	–	5.0	0.8	–	2.25	86.95
NLC C5-TOC	–	2.5	–	–	2.5	0.4	–	2.37	92.23
NLC C10-TOC	–	5.0	–	–	5.0	0.8	–	2.25	86.95
NLC P5-TOC	–	–	2.5	–	2.5	0.4	–	2.37	92.23
NLC P10-TOC	–	–	5.0	–	5.0	0.8	–	2.25	86.95
NLC S5-TOC	–	–	–	2.5	2.5	0.4	–	2.37	92.23
NLC S10-TOC	–	–	–	5.0	5.0	0.8	–	2.25	86.95
NLC T10-RA	5.0	–	–	–	4.98	–	0.02	2.25	87.75

^a^TOC: α-tocopherol; ^b^RA: retinoic acid.

### Photon correlation spectroscopy (PCS) analysis

Submicrometer particle analysis was performed using a Zetasizer Nano S90 device (Malvern Instruments, Malvern, England) equipped with a 5 mW helium neon laser with a wavelength output of 633 nm. The glassware was cleaned of dust by washing with detergent and rinsing twice with water for injections. The measurements were made in triplicate at 25 °C at an angle of 90°, and the data were interpreted using the “CONTIN” method [38].

### Cryogenic transmission electron microscopy (cryo-TEM) analysis

The samples were vitrified as previously described [39]. The vitrified specimen was transferred to a Zeiss EM922 Omega transmission electron microscope for imaging using a cryoholder (CT3500, Gatan). The temperature of the sample was kept below −175 °C throughout the examination. The specimens were examined with doses of about 1000–2000 e/nm^2^ at 200 kV. The images were digitally recorded by a CCD camera (Ultrascan 1000, Gatan) using an image processing system (GMS 1.9 software, Gatan). In addition, the size distribution of the nanoparticles was performed by measuring 1000 nanoparticles for each cryo-TEM image by the digital analyzer ImageJ 1.48v.

### Small-angle X-ray scattering (SAXS) measurements

Small-angle X-ray scattering (SAXS) experiments were performed at the SAXS BM29 beamline of the European Synchrotron (ESRF) in Grenoble, France. NLC samples were filled in glass capillaries. The experiments were performed at 30 and 37 °C, both in the presence and absence of TOC and RA. The investigated *Q*-range (*Q* = 4π sinθ/λ, where 2θ is the scattering angle and λ is the X-ray wavelength) was from 0.01 to 0.5 Å^−1^, the wavelength used was 0.99 Å. The sample exposure time was 160 s, which ensured enough statistical accuracy without degrading the samples by radiation. The Bragg peaks observed were indexed considering the possible symmetries commonly observed in lipid systems (lamellar, hexagonal or cubic) [40]. Accordingly, from the averaged spacing of the observed peaks the unit cell dimension of the phase was calculated.

### Encapsulation efficiency and loading capacity of lipid nanoparticles

The encapsulation efficiency (EE) and loading capacity (LC) of TOC and RA in NLCs were determined as previously described [41]. A 0.5 mL aliquot of each NLC batch was loaded into a centrifugal filter (Microcon centrifugal filter unit YM-10 membrane, NMWCO 10 kDa, Sigma-Aldrich, St. Louis, MO, USA) and centrifuged (Spectrafuge™ 24D Digital Microcentrifuge, Woodbridge, NJ, USA) at 8,000 rpm for 20 min. The amount of drug was determined after dissolving the lipid phase with a known amount of methanol (1:10 v/v) for 2 h under stirring. The TOC and RA content was analyzed after filtration by high-performance liquid chromatography (HPLC) using a Knauer Eurospher II RP C18 column (Knauer, Germany) (15 × 0.4 cm) stainless steel packed with 5 µm particles, eluted at room temperature with different mobile phases. Samples of 50 µL were injected through the rheodyne injector system fitted with a 50 µL fixed loop and compared with standards of known concentration. In the case of TOC, the mobile phase was methanol, and the flow rate was 1 mL/min at 295 nm, while for RA, acetonitrile/methanol/methylene chloride (70:15:15, v/v) was employed, with a flow rate of 1 mL/min at 325 nm. The analyses were conducted in triplicate. EE and LC were determined using Equation 1 and Equation 2

[1] EE=L/T×100

[2] $$\text{LC}=L/T_{\text{lipid}\;\text{phase}}\times 100$$

where *L* is the amount of drug effectively present within the nanoparticles, *T* stands for the total amount of drug initially added to the lipid phase and *T*_lipid phase_ is the total weight of lipid phase in the formulation. Determinations were performed six times in independent experiments and the mean values ± standard deviations were calculated.

### Stability studies

After production, the nanoparticles were stored in glass containers at 25 °C for 6 months [42]. To assess the physical and chemical stability, particle size analysis and TOC encapsulation efficiency were periodically evaluated by PCS and HPLC, respectively, as above reported.

### Western blot analysis for HO-1 and HO-2 protein

#### Cytotoxicity determination

Experiments were carried out to assess the range of NLC T10-TOC, NLC C10-TOC, NLC P10-TOC and NLC S10-TOC concentrations that are nontoxic for cells. Briefly, human immortalized keratinocytes (HaCaT) were treated for 24 h with the different NLC formulations at various TOC concentrations, ranging from 25 to 200 µM. Cytotoxicity was evaluated by spectrophotometric quantification of the LDH released in culture medium, using a commercial kit (Sigma-Aldrich, Merck, Darmstadt, Germany), as previously described [43].

#### Human skin explant (HSE) culture

Skin explants were prepared from the superfluous skin of healthy adult donors (18–60 years old). Breast or abdominal tissue specimens were obtained from patients undergoing plastic surgery. Skin biopsies (12 mm punches) were cultured into standard 6-well plates in contact with culture medium at 37 °C in 5% CO_2_ humidified air. The culture medium was Dulbecco’s Modified Eagle Medium (DMEM) with 1% antibiotic-antimycotic solution (10,000 units penicillin, 10 mg streptomycin and 25 μg amphotericin B – Sigma-Aldrich, Germany) and 1% ʟ-glutamine (Sigma-Aldrich, Germany) [43]. After 1 day in culture, the medium was changed and HSEs were topically treated with 50 µL of NLC T10 and NLC T10-TOC for 24 h.

#### Cigarette smoke (CS) exposure

After 24 h of treatment, the HSEs were exposed for 30 minutes to CS generated by burning one research cigarette (12 mg tar, 1.1 mg nicotine) using a vacuum pump, as previously described [44]. Control HSEs were exposed to filtered air. After exposure, the explants were incubated in fresh media at 37 °C in a humidified 5% CO_2_ atmosphere for 24 h.

#### Protein extraction

Samples for Western blot analysis were washed in PBS and frozen in liquid nitrogen. The biopsies were extracted in ice-cold conditions using a tissue protein extraction reagent (T-PER buffer, Thermo Fisher Scientific, MA, USA) added consisting of protease and phosphatase inhibitor cocktails (Sigma, Milan, Italy), using a bead-based homogenizer at 12400 rpm at 4 °C for 15 min. The protein concentration was measured by the Bradford method (BioRad, CA, USA) [40].

#### Western blot analysis

The samples (25 µg protein) were loaded onto 10% sodium dodecyl sulfate polyacrylamide gel (SDS-PAGE) and then transferred onto nitrocellulose membranes. Blots were blocked in PBS containing 0.5% Tween 20 and 5% not-fat milk (BioRad). The membranes were incubated overnight at 4 °C with the appropriate primary antibody HO-1 (Abcam, Cambridge, UK). The membranes were then incubated with horseradish peroxidase conjugated secondary antibody for 1 h at RT, and the bound antibodies were detected in a chemiluminescent reaction (ECL, BioRad). Chemiluminescence was detected on a ChemiDoc imager (BioRad) [45]. The blots were reprobed with β-actin as the loading control. Images of the bands were digitized, and the densitometry of the bands was performed using ImageJ software [46].

#### Statistical analysis

For each of the variables tested, two-way analysis of variance (ANOVA) was used. A significant result was indicated by a *p* value <0.05. All the results are expressed as mean ± SD of 6 determinations for nanoparticle characterization experiments and 3 determinations obtained in 3 independent experiments for in vitro cultured cells tests.

## Results and Discussion

### Effect of lipid composition on nanoparticle macrostructure

In order to obtain a nanoparticulate system suitable for cutaneous administration of antioxidants, different lipid compositions have been considered, as reported in Table 1 and Table 2. The selection of the lipid composition has been performed by choosing nottoxic, commercial lipids with similar chemical composition and different carbon chain lengths on the basis of our expertise concerning tristearin and caprylic/capric triglycerides (miglyol) [39]. Namely, SLNs were produced based on the use of solid di- or tri-glycerides, with chain lengths ranging between 18 and 21 carbon atoms, while for NLC production, the same solid lipids were employed in mixture (1:1 w/w) with the liquid caprylic/capric triglycerides (miglyol), characterized by C_8_–C_10_ chains.

With regard to surfactant concentration, higher poloxamer amounts, namely 3 and 4% w/w with respect to the aqueous phase, have been tested. However, the increase of poloxamer led to foam formation during the preparation, which caused irregular and inhomogeneous formulations; thus 2.5% w/w of poloxamer was used.

The hot homogenization method followed by ultrasound [39] lead to production of milky and homogeneous dispersions. Immediately after cooling, in most cases, a small amount of coalesced lipid phase appeared on the surface of the dispersion. The weight of this agglomerate, spanning between 0 and 4.65% by weight with respect to the total amount of the lipid phase, was a function of the lipid composition. Indeed, both the amount and the type of lipid phase appear to influence the agglomerate formation. Particularly, the longer the lipid chain, the higher the agglomerate weight according to the following trend: compritol (2 C_21_ chains) > tristearin (3 C_18_ chains) > precirol (2 C_18_ chains) > suppocire (3 C_10_–C_18_ chains). Specifically, in the latter case, the agglomerate was almost absent (Table 4). In addition, the extent of agglomeration was lower for NLC, probably due to the presence of the liquid lipid.

**Table 4:** Dimensional characteristics of SLNs or NLCs and the presence of agglomerates.

Preparation	Z-average, *D*_z_ (nm)	Polydispersity index	Agglomerate^a^ (%)

SLN T5	148.6 ± 74.5	0.35 ± 0.11	2.86 ± 0.04
SLN T10	164.9 ± 9.4	0.33 ± 0.04	4.65 ± 0.03
SLN C5	244.5 ± 26.5	0.36 ± 0.01	3.43 ± 0.02
SLN C10	488.9 ± 25.3	0.32 ± 0.04	3.77 ± 0.04
SLN P5	245.5 ± 31.9	0.31 ± 0.03	2.48 ± 0.03
SLN P10	453.1 ± 9.3	0.39 ± 0.02	2.99 ± 0.04
SLN S5	220.9 ± 15.6	0.37 ± 0.06	1.13 ± 0.03
SLN S10	201.8 ± 82.1	0.39 ± 0.06	0.60 ± 0.04

NLC T5	122.6 ± 34.2	0.32 ± 0.07	2.14 ± 0.02
NLC T10	127.9 ± 29.1	0.29 ± 0.03	2.54 ± 0.01
NLC C5	160.2 ± 25.5	0.29 ± 0.03	3.15 ± 0.02
NLC C10	136.8 ± 51.5	0.24 ± 0.04	3.25 ± 0.01
NLC P5	148.1 ± 29.5	0.22 ± 0.10	1.51 ± 0.01
NLC P10	159.8 ± 33.7	0.30 ± 0.08	1.84 ± 0.02
NLC S5	131.3 ± 30.5	0.31 ± 0.05	0 ± 0.01
NLC S10	136.1 ± 13.5	0.29 ± 0.07	0 ± 0.01

^a^Loss of lipids (lipid phase) due to the partial coalescence of the lipid phase during the formation of the O/W emulsion. % refers to the weight of the lipid phase. Data represent the mean ± SD of 6 independent experiments.

### Effect of lipid composition on nanoparticle size distribution

**Figure 1:**
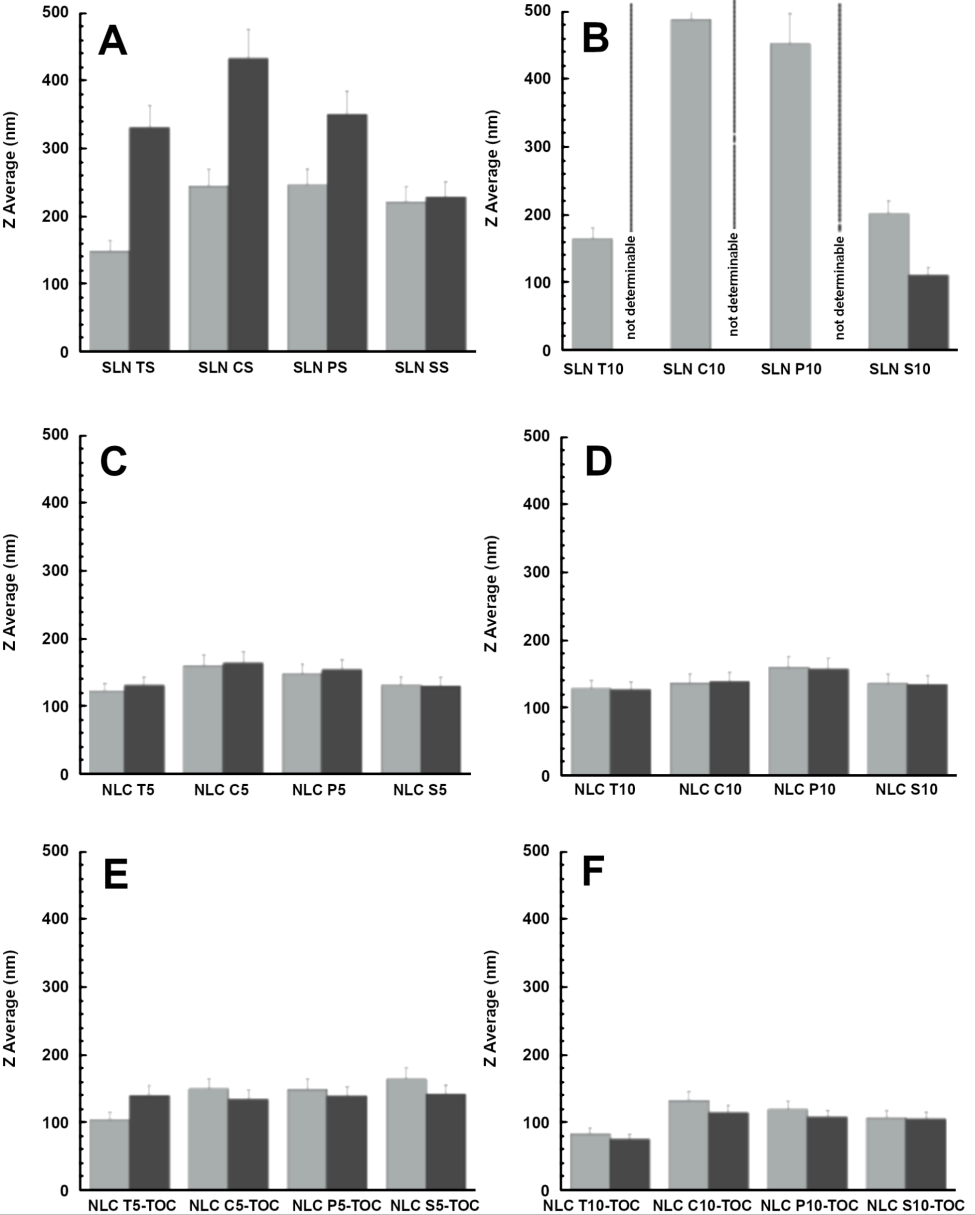
Variation of the Z-average mean diameters, *D*_z_, of SLNs (A, B) and NLCs (C, D) produced in the absence of antioxidants and NLC loaded with TOC (E, F) evaluated at 1 (light grey) and 90 (grey) days after nanoparticle production. In the case of SLN T10, SLN C10 and SLN P10, mean diameters were not measurable by PCS 90 days after production.

The SLN and NLC dimensions, measured by PCS and expressed by the Z-average, *D*_z_, are reported in Figure 1 and Table 4. In the case of SLN with 5% lipid phase concentration, mean diameters were comprised between 148 and 245 nm, with some differences due to the lipid composition. The doubling of the lipid phase concentration, however, induced an increase of the mean diameter, especially in the case of SLN P10 and SLN C10, whose Z-average reached almost 500 nm. In the case of NLC, the lipid phase composition scarcely affected the mean diameter, ranging between 125 and 160 nm both for 5% and 10% of lipid phase. The polydispersity index was always below 0.39, with smaller values in the case of NLCs.

The Z-average mean diameters of SLNs and NLCs stored at 25 °C were measured after 3 months from production. In the case of SLNs, the mean diameter dramatically increased, as reported in Figure 1A and 1B, especially for SLN T10, SLN C10 and SLN P10, reaching values undetectable by PCS, where instead, the mean diameter of SLN S5 and SLN S10 did not improve. This behavior can be attributed to the lipid phase containing SLN that influences both agglomerate and the mean diameter of the nanoparticles. On the contrary, the NLCs maintained their mean diameters almost unvaried, irrespectively of the lipid phase type and concentration (Figure 1C and 1D).

In order to avoid agglomeration phenomena and to control the mean size, only NLCs have been considered for antioxidant loading.

### Production and characterization of NLCs containing antioxidants

To produce antioxidant-containing NLCs, different amounts of TOC and RA were loaded in NLCs, as reported in Table 3. Particularly, since TOC is practically insoluble in water (logP 8.84), it was directly added to caprylic/capric triglycerides (miglyol) in order to improve its solubility (reaching 16 mg/mL) before addition of solid lipids [47]. The doubling of the lipid phase concentration enabled to doubling the amount of TOC loading. The macroscopic characteristic of NLCs containing TOC was milky and homogeneous, similar to the empty NLCs. Both the presence of agglomerates and the mean size of NLCs containing TOC were lower with respect to their empty counterparts (Table 5). This trend suggest that TOC could contribute to stabilize the interface between the lipid and the aqueous phase, leading to smaller droplets and finally to smaller nanoparticles. The agglomerate presence was more evident in the case of compritol and absent in the case of suppocire, as in the case of empty NLCs, while mean dimensions were inversely proportional to the amount of lipid phase and TOC. As observed in the case of the empty counterparts, Z-average mean diameters, *D*_z_, of antioxidants containing NLC stored at 25 °C for 3 months were almost unvaried (Figure 1E and 1F). Particularly, NLC T10-TOC displayed the smallest mean diameter, even after 3 months.

**Table 5:** Dimensional characteristics, agglomeration and encapsulation parameters of antioxidant-containing NLCs.

NLC preparation	Z-average, *D*_z_ (nm)	Polydispersity index	Agglomerate^a^ (%)	Encapsulation efficiency^b^	Loading capacity^c^

NLC T5-TOC	104.5 ± 32.0	0.33 ± 0.11	1.22 ± 0.02	90.96 ± 1.3	7.27 ± 0.1
NLC T10-TOC	82.8 ± 10.7	0.36 ± 0.05	1.24 ± 0.01	90.69 ± 2.8	7.25 ± 0.2
NLC C5-TOC	149.4 ± 36.9	0.22 ± 0.03	2.25 ± 0.01	95.61 ± 1.5	7.64 ± 0.2
NLC C10-TOC	132.7 ± 51.3	0.34 ± 0.05	3.17 ± 0.02	79.15 ± 2.5	6.33 ± 0.1
NLC P5-TOC	149.5 ± 30.3	0.30 ± 0.02	1.11 ± 0.01	93.58 ± 1.7	7.48 ± 0.1
NLC P10-TOC	118.5 ± 31.9	0.30 ± 0.05	1.12 ± 0.02	90.99 ± 2.2	7.27 ± 0.2
NLC S5-TOC	164.6 ± 21.7	0.29 ± 0.06	0.00 ± 0.01	88.16 ± 1.3	7.05 ± 0.1
NLC S10-TOC	106.1 ± 24.2	0.29 ± 0.07	0.00 ± 0.01	60.72 ± 2.1	4.85 ± 0.1

NLC T10-RA	98.4 ± 20.2	0.27 ± 0.12	2.52 ± 0.01	67.24 ± 0.8	0.16 ± 0.0

^a^Loss of lipids (lipid phase) due to the partial coalescence of the lipid phase during the formation of the O/W emulsion. After cooling the coalesced lipid phase appeared as a small flake floating on the surface of the NLC dispersion. ^b^Percentage (w/w) of drug in the whole dispersion with respect to the total amount used for the preparation. ^c^Percentage (w/w) of drug within nanoparticles as compared to the amount of lipid used for the preparation. Data represent the mean ± S.D. of 6 independent experiments.

Due to the encouraging results obtained using tristearin 10%, RA was loaded into NLC T10. Due to its poor water solubility (logP 6.3), as in the case of TOC, RA was added to caprylic/capric triglycerides (miglyol), improving its solubility to 4 mg/mL [48]. In the case of NLC T10-RA, despite the small mean diameter (98 nm), the agglomeration phenomenon was more noticeable as compared to NLC T10-TOC (Table 5).

**Figure 2:**
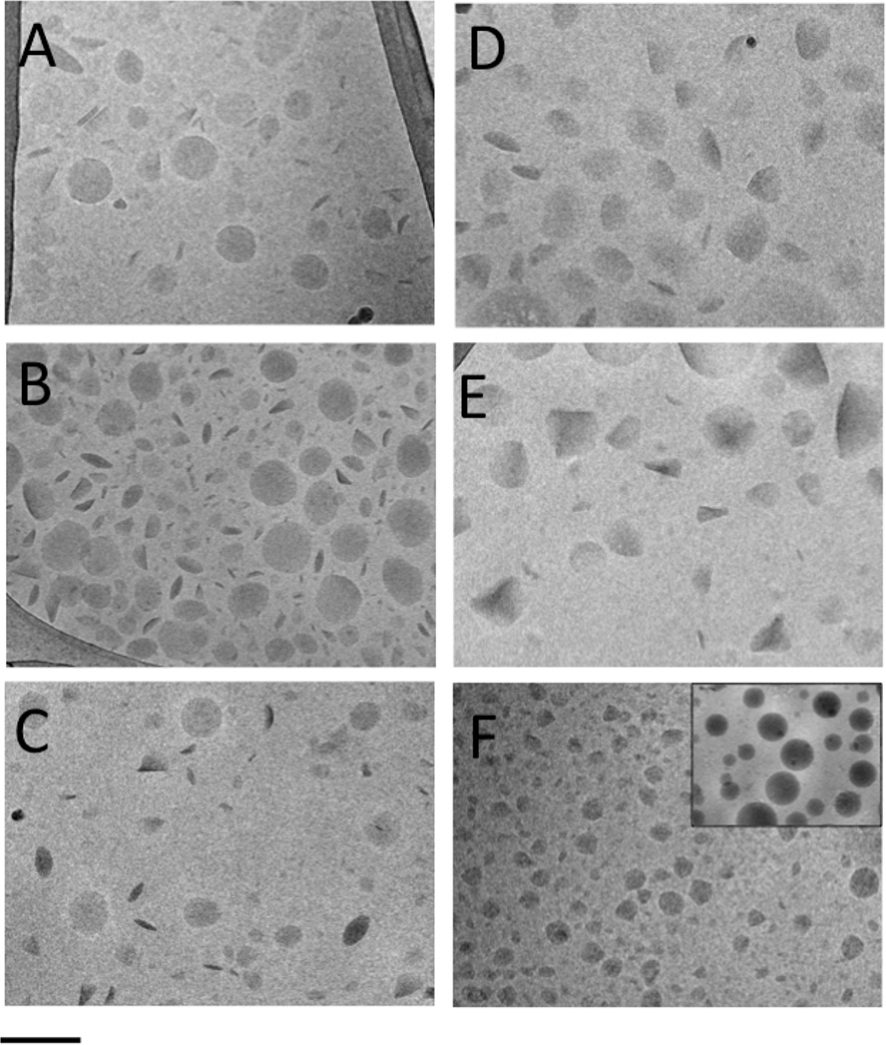
Cryo-TEM images of NLC T10 (A), NLC T10-TOC (B), NLC T10-RA (C), NLC C5-TOC (D), NLC P5-TOC (E), NLC S5-TOC (F). The scale bar below corresponds to 200 nm in panels A–E and 300 nm in panel F.

The NLC morphology was investigated by cryo-TEM and a few images are reported in Figure 2. In general, the NLC shape appears discoid in the top view, or more electron-dense and rod-like in the edge-on view. In the case of tristearin-based NLCs, the shape was roundish, both for empty (Figure 2A) and antioxidant-loaded NLC T10 (Figure 2B and C). In the case of compritol (Figure 2D) and precirol (Figure 2E) based NLCs, ovoid and triangular structures were observed. At last, in the case of suppocire NLCs, besides the presence of some irregular structures (Figure 2F), spherical structures were detected (inset of Figure 2F), resembling vesicles rather than to solid particles.

**Figure 3:**
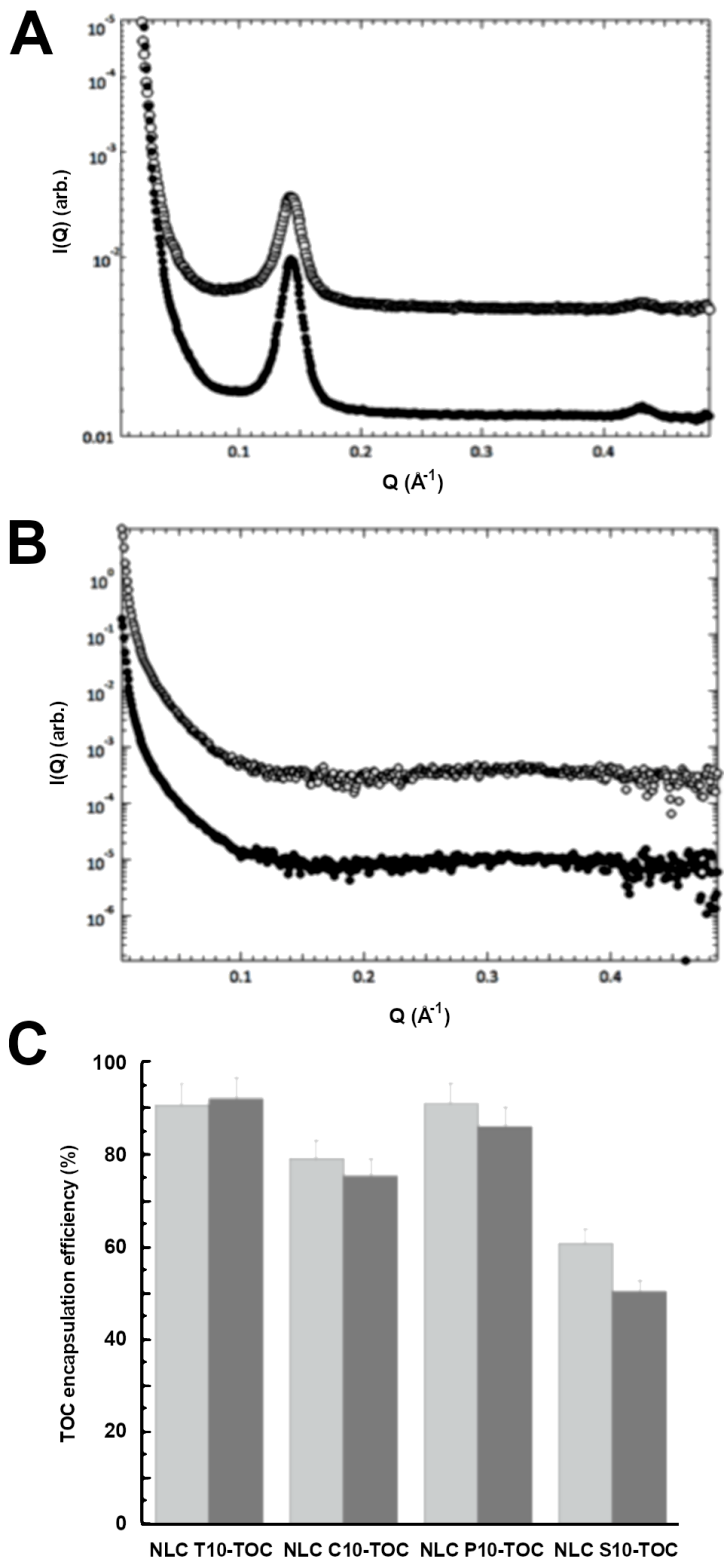
SAXS profiles observed for A: NLCT5-TOC (open symbol) and NLCT10-TOC (closed symbol), B: NLCS5-TOC (open symbol), and NLCS10-TOC (closed symbol). C: TOC encapsulation efficiency in the indicated NLC, evaluated at 1 (light grey) and 90 (grey) days after production.

The inner morphology of the NLCs was further characterized by SAXS [39–40]. In particular, SAXS experiments were performed on NLC samples prepared by using tristearin or suppocire both in the presence and in the absence of TOC and RA. By way of illustration, Figure 3 shows the low-angle diffraction profiles obtained as a function of the lipid phase concentration from tristearin-based NLCs (top graph, A) and from suppocire-based NLCs (middle graph, B), both containing TOC. The presence of Bragg peaks in the NLC T5-TOC and NLC T10-TOC samples shows that the inner structure of the NLC at 30 °C depends on the used lipid: tristearin guarantees the presence of an ordered structural organization inside the NLC, while suppocire is not able to preserve such an organization. According to the cryo-TEM findings, vesicles rather than nanoparticles probably form in this condition.

The analysis of the position of the peaks observed in tristearin-based NLCs allowed the identification of the internal structural organization for NLC T5-TOC and NLC T10-TOC: because the spacing ratios scale as 1:2:3.., a lamellar organization was derived [34–35]. The corresponding unit cell dimension, which in a lamellar organization is the repeated distance between two lamellae, was 43.9 Å. It should be noted that similar results were obtained from empty tristearin-based NLCs: the packing of the lamellae in the nanoparticle inner region is not affected by the presence of the antioxidant. The scattering profiles obtained from NLC T5-RA and NLC T10-RA were very similar (data not shown), confirming the described behavior.

### Encapsulation of antioxidants in NLCs

The influence of the NLC lipid composition on the capability to incorporate antioxidants was studied by evaluating the EE and LC. The values reported in Table 5 and Figure 3C evidenced that in the case of tristearin or precirol based NLCs, the EE of TOC was >90%, irrespective of the lipid phase concentration. In the case of NLC C10-TOC, both a decrease in the EE value and an increase in agglomeration were detected with respect to NLC C5-TOC, suggesting that the doubling of the lipid concentration promoted agglomeration of the lipid phase, partially avoiding TOC encapsulation within the nanoparticles. This hypothesis was corroborated by disaggregation and HPLC analysis of the lipid phase agglomerate, revealing the presence of 13% w/w TOC with respect to the total amount used for NLC production.

The lowest EE values were found in the case of NLC S10-TOC and NLC T10-RA. In the case of suppocire, TOC EE values decreased from 88 to 60% by doubling the lipid phase concentration, suggesting that the presence of vesicles instead of nanoparticles prevented high loading of the antioxidant within their structure.

Regarding NLC T10-RA, as for NLC C10-TOC, an amount of antioxidant (11%) was found within the agglomerate of the lipid phase, justifying the reason for the low EE value of RA. LC values of NLCs containing TOC were between 4.85 and 7.64%, whilst in the case of RA, the LC was only 0.16% due to the lower amount of RA employed for NLC production (0.05 mg/100 mg lipid phase, instead of 8 mg/100 mg lipid phase used in the case of TOC).

In order to detect the capability of NLC to control the encapsulation of antioxidants under storage, the EE values were evaluated for 90 days (Figure 3C). Particularly, NLCs containing 10% lipid phase were selected due to their marked dimensional stability.

The TOC EE values were almost unvaried in the case of NLC T10-TOC – they slightly decreased in the case of NLC C10-TOC and NLC P10-TOC, whilst the decrease was more evident in the case of NLC S10-TOC, passing from 60 to 48%*.* It can be hypothesized that the prevalence of vesicles in NLC S10-TOC, instead of more structured carriers, hindered the TOC encapsulation. Lastly, in the case of NLC T10-RA, RA encapsulation dramatically decreased –the EE value halved one month after production (data not shown). Due to their poor stability, NLC T10-RA samples were not considered for further studies.

### Cytotoxicity of NLCs containing TOC

As the produced formulations are intended for topical administration on the skin, experiments on human keratinocytes were conducted in order to test the cytotoxicity of NLC T10-TOC, NLC C10-TOC, NLC P10-TOC and NLC S10-TOC. The LDH release in the media was assessed 24 h after TOC treatment at the concentrations of 25, 50, 100 and 200 µM.

**Figure 4:**
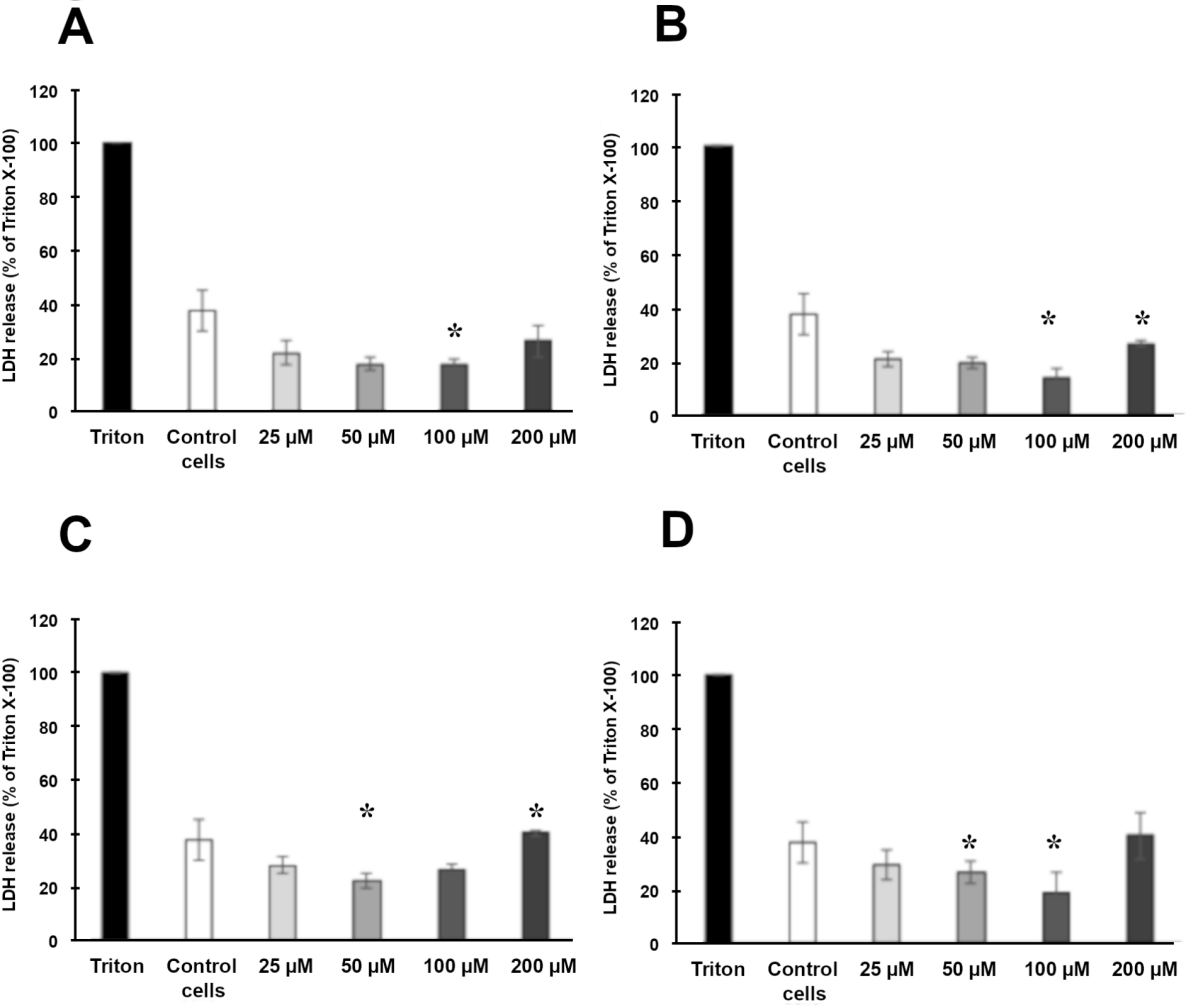
Cytotoxicity of NLC T10-TOC (A), NLC C10-TOC (B), NLC P10-TOC (C) and NLC S10-TOC (D) evaluated by LDH release from HaCaT cells in the media after 24 h of treatment. Data are expressed as percentage LDH release as compared to the maximum release of LDH from Triton-X100-treated cells. Data are given as mean ± SD, representative of three independent experiments with at least three technical replicates each time. * indicates statistically significant difference to untreated control cells (unpaired *t*-test, *p* < 0.01).

As shown in Figure 4, no NLC cytotoxicity was observed with respect to control cells and no significant difference among the different NLCs was noticed, confirming the biocompatibility of the components.

Due to the obtained results, NLC T10-TOC samples were selected for further ex vivo studies. Indeed, this kind of NLC displayed better physico-chemical properties with respect to NLC based on different lipid compositions, being able to longer maintain the size and the EE of TOC.

### Antioxidant effect of NLCs containing TOC

Following the results obtained in the 2D cell model, the study of the protective effect of NLC T10-TOC was carried out on HSE.

**Figure 5:**
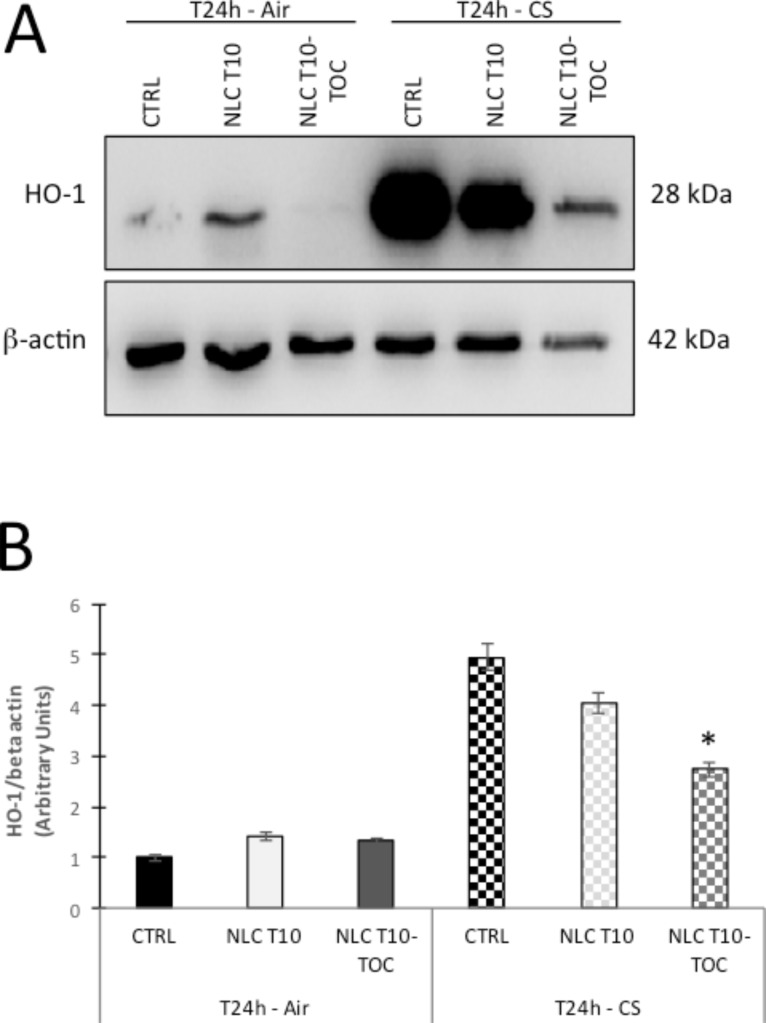
Effect of cigarette smoke (CS) on heme-oxygenase (HO-1) expression evaluated on human skin explants (HSE) treated with NLCT 10 or NLCT 10-TOC, exposed to air or CS for 30 min and harvested after 24 h. A) Representative Western blot analyses of HO-1 protein expression (with the respective β-actin controls). B) Mean expression of HO-1 as a ratio of β-actin. The results are shown as the mean of three experiments. * *p* < 0.05 with respect to the control.

CS contains many components able to elicit oxidative stress, which can induce the cytoprotective enzyme heme oxygenase (HO-1). An HO-1 increase promotes protection against inflammation and/or cell death induced by CS [49–50]. In order to evaluate the effect of NLC T10-TOC in preventing damage caused by CS, the HO-1 expression was evaluated on HSE cultures exposed to CS or to air for 24 h. Namely HO-1 has been determined by Western blot analysis, quantified by densitometry and normalized to the beta-actin level for each sample (Figure 5A). The mean relative density ratios of three experiments are shown in Figure 5B. As depicted, the expression of the HO-1 protein level is significantly induced by the CS exposure because of the ability of this outdoor stressor to promote oxidative-related cellular modifications to the skin [39]. On the other hand, HO-1 levels in skin explants treated with NLC T10-TOC and exposed to CS were dramatically and significantly prevented (47% decrease, *p* < 0.001 vs control).

These results suggest that NLC T10-TOC can effectively reduce the induction of cutaneous HO-1, which is a sensor of tissue stress, suggesting the ability of this topical application to prevent CS-induced skin damage. Further studies will be required to investigate the dose and type-dependent manner of action of TOC loaded in NLCs with respect to an unloaded TOC solution.

## Conclusion

This work has underlined the importance of technological screening in the design of a nanoparticulate lipid dosage formulation. Notably, dimensional and morphological characterization of nanoparticles should be performed at different durations of time after production. This investigation has demonstrated that the type and concentration of the lipid phase affect the physico-chemical stability of nanoparticles. The NLC T10-TOC sample that was selected by the preformulation study deserved further in vitro and in vivo studies. Indeed, supplementary studies will be performed to investigate the activity of hydrophilic antioxidant molecules, such as ascorbic acid and *N*-acetyl-cysteine, loaded in NLCs and in comparison with conventional “non-nano” formulations. In addition, since some authors have demonstrated that CS induces depletion of some essential vitamins, such as TOC and RA [23], it should be interesting to evaluate the suitability of NLC T10-TOC as an oral antioxidant supplement.
